# Predictive models for charitable giving using machine learning techniques

**DOI:** 10.1371/journal.pone.0203928

**Published:** 2018-10-03

**Authors:** Leily Farrokhvar, Azadeh Ansari, Behrooz Kamali

**Affiliations:** Department of Industrial and Management Systems Engineering, West Virginia University, Morgantown, West Virginia, United States of America; University of Zaragoza, SPAIN

## Abstract

Private giving represents more than three fourths of all U.S. charitable donations, about 2% of total Gross Domestic Product (GDP). Private giving is a significant factor in funding the nonprofit sector of the U.S. economy, which accounts for more than 10% of total GDP. Despite the abundance of data available through tax forms and other sources, it is unclear which factors influence private donation, and a reliable predictive mechanism remains elusive. This study aims to develop predictive models to accurately estimate future charitable giving based on a set of potentially influential factors. We have selected several factors, including unemployment rate, household income, poverty level, population, sex, age, ethnicity, education level, and number of vehicles per household. This study sheds light on the relationship between donation and these variables. We use Stepwise Regression to identify the most influential variables among the available variables, based on which predictive models are developed. Multiple Linear Regression (MLR) and machine learning techniques, including Artificial Neural Networks (ANN) and Support Vector Regression (SVR) are used to develop the predictive models. The results suggest that population, education level, and the amount of charitable giving in the previous year are the most significant, independent variables. We propose three predictive models (MLR, ANN, and SVR) and validate them using 10-fold cross-validation method, then evaluate the performance using 9 different measuring criteria. All three models are capable of predicting the amount of future donations in a given region with good accuracy. Based on the evaluation criteria, using a test data set, ANN outperforms SVR and MLR in predicting the amount of charitable giving in the following year.

## Introduction

Charities are Non-Profit Organizations (NPO) focused on humanitarian and social issues [1]. The NPOs are listed as tax-exempt organizations which cannot benefit people or other corporations [2]. According to the National Center for Charitable Statistics, there are 1,406,820 tax-exempt organizations in the United States, including 945,415 public charities. Charitable contributions include cash and non-cash gifts [3]. Over two-thirds of households making a significant portion of charitable giving in the United States announce their own giving amount [4]. Americans recently assigned approximately 2% of their disposable income to charitable goals in 2010, a value unchanged over 5 decades before that [5]. Over three-fourths of all contributions come from private or individual donors earn more than 2% of total U.S. Gross Domestic Product (GDP). Their contribution is essential to the nonprofit sector of the U.S. economy [5].

The National Center for Charitable Statistics stated at there were $1.59 trillion in total revenues and $1.49 trillion in total expenses reported by public charities in 2011 [6]. Contributions and government grants made up 22% of the total revenue while program service revenues and other resources made 72% and 6% of the revenue, respectively [1]. Charitable giving has had an upward trend since 1975 and in 2014, it exceeded the peak level right before the recession in 2008 [5]. It was estimated that charitable giving would increase to $358 billion in 2015 from $298 billion in 2011. Individual donors are the most common sources of charitable giving [7]. There is growing competition over time, money, and resources among non-profit organizations, so there is a need to predict donations [8]. It has always been debated how and when donors decide to donate, and many factors are identified in the literature that can affect decisions [9].

The factors that have been reported to drive charitable giving are awareness of need [10], solicitation [11,12], mode of communication [13], donor characteristics [14] including age [4,15, 16], sex [17], ethnicity [4,17], personal income [4,15] and tax itemizing [15], education [4,15], volunteerism/civic-minded donor [4,18], moral norms/values & guilt [11, 12], religiosity [4, 16, 19, 20], obligation (wealthy) [21] or lack of family need [11], and attitudes toward charitable organizations [22], historical data on donation [16], size of request [23], altruism [10, 11], reputation of individual/charity [24], psychological benefits [10], and efficacy [10]. Bekkers and Wiepking [25] performed an extensive literature review on how age, education, religion, and solicitation are correlated with the amount of charitable giving. They stated that most of the previous works had found that education and age have positive relationship with the amount of donation and investigated the effects of different characteristics of religion and solicitation on the giving amount. Wiepking and Bekkers [26] completed their literature review and investigated how sex, family composition, and income are related to charitable giving. They claimed that there is strong evidence showing a positive relationship between income and charitable giving value.

In this manuscript, we aim to develop models capable of predicting levels of charitable giving using measurable and readily available variables describing donor characteristics for different zip codes that are most closely associated with charitable giving on a national level. We validated the models using a U.S. nationwide dataset and analyzed the results.

## Methodology

### Giving and demographic data

In this section, we describe the process of collecting and preparing the initial set of variables that are used to identify the factors with the most predictive value for the amount of charitable giving. The following demographic data from 2010 U.S. census is gathered for a sample of 9410 U.S. zip codes, each of which includes at least one NPO with charitable contributions in 2014 and 2015: (a) % unemployment, (b) median household income, (c) % of persons living at/below the poverty line, (d) population, (e) sex, (f) age, (g) ethnicity (% non-whites), (h) education (% college graduates), and (i) % households with 2 vehicles or more. This data was collected using Zip Atlas (www.zipatlas.com), which is a structured collection of zip code, area code, city and state demographic, social, and economic profiles. We also included the previous year’s total contribution amount for each zip code as a potential predictive variable. The data regarding charitable contributions to NPOs was obtained by reviewing U.S. federal tax forms (990 and 990EZ) for two consecutive years (2014 and 2015). All the organizations that have some portion of their income exempted from tax are required by the IRS to fill out one of these forms, including organizations that received charitable contributions and grants. These forms are publicly available on the Internal Revenue Service website (IRS; www.irs.gov). Overall, about 25,882 organizations were reviewed for 2014 and 28,517 for 2015. To have consistent scope for all the data elements, we aggregated the contribution data at the zip code level. All the variables are defined in Table 1. The values or ratios are all collected for each specific zip code.

**Table 1:** Variable definitions.

**Variables**	**Definition**
**Unemployment Rate (%)**	Percentage of unemployed individuals to all individuals currently in the labor force
**Average Household Income**	Combined incomes of all people sharing a particular household
**Poverty Level (%)**	Percentage of the number of people (in a given age group) whose income falls below the poverty line
**Average Population Age**	The age that divides a population into two numerically equal groups
**Population**	Number of people living in the zip code
**Whites (%)**	Percentge of white Americans to all population
**College Graduates (%)**	The ratio of the number of people with college degree to all population
**Male/Female Ratio**	The ratio of male to female in the population
**Households with 2+ Cars (%)**	Percentage of number of houses with 2 or more cars
**Giving**	The amount of donation (charitable giving) a NPO receives

As the donation data set is skewed, Tukey’s [27] outlier detection method was used to identify the outliers. To be more conservative, all data points falling outside the 3 Interquartile Range (IQR) were identified as outliers and Winsorized [27]. Winsor proposed to replacing the value of the potential outliers by the highest value that is not considered an outlier in the data set instead of removing them. To have the same scale for all the variables and coeffiecients, all the variables were normalized using equation.

$$X_{N}=\frac{X-Min\{X\}}{Max\{X\}-Min\{X\}}$$ (1)

In equation, *X* and *X*_*N*_ denote the original and normalized data, respectively. Each entity in the data set is normalized using its own minimum and maximum values shown by *Min*{*X*} and *Max*{*X*}, respectively.

In this pre-processing step, aggregated contribution data associated with 131 zip codes were Winsorized as they were beyond the 3 IQR limits. All 9410 rows of data were then normalized using equation. Table 2 summarizes the descriptive statistics on all the variables including 9 demographic variables and 2014 and 2015 donation data.

**Table 2:** Descriptive statistics for all variables.

**Variables**	**Mean**	**Std. Dev.**	**Min**	**Max**
**Unemployment Rate (%)**	0.053	0.040	0	1
**Average Household Income**	47,350.080	19,100.640	5,787	196,298
**Poverty Level (%)**	0.078	0.070	0	1
**Average Population Age**	37.094	5.276	16.300	75
**Population**	19,145.170	15,666.840	5	114,124
**Whites (%)**	0.828	0.186	0.006	1
**College Graduates (%)**	0.264	0.159	0	0.945
**Male/Female Ratio**	0.974	0.138	0.250	5.140
**Households with 2+ Cars (%)**	0.537	0.156	0	1
**Giving 2014**	42,836.180	64,130.710	0	1,320,216
**Giving 2015**	44,090.530	65,641.760	0	1,470,209
**Giving 2015 (Winsorized)**	42,611.990	56,739.420	0	249,165

The correlation matrix in Table 3 shows Pearson’s correlation coefficients between all the variables, including the dependent variable. Pearson’s correlation coeficients show how well these variables are linearly related to each other and the output.

**Table 3:** Correlation matrix on dependent variables.

Variables	*Unemp*. Rate (%)	*Income*	*Pov*. %	*Avg*. *Pop*. *Age*	*Pop*.	*Whites* %	*Coll*. *Grad*.	*M/ F* Ratio	*H/holds 2+ Cars* %	*Giving* 2014	*Giving 2015*
**Unemp. Rate (%)**	1	-0.46	0.67	-0.24	0.07	-0.45	-0.30	0.09	-0.50	0.01	0.02
**Income**		1	-0.63	0.14	0.05	0.22	0.72	-0.06	0.58	0.08	0.08
**Pov. (%)**			1	-0.30	0.08	-0.57	-0.42	0.05	-0.59	0.02	0.02
**Avg. Pop. Age**				1	-0.33	0.40	0.08	-0.16	-0.03	-0.02	-0.03
**Pop.**					1	-0.39	0.11	-0.12	-0.11	0.18	0.19
**Whites (%)**						1	0.09	-0.04	0.43	-0.07	-0.08
**Coll. Grad. (%)**							1	-0.08	0.15	0.19	0.19
**M/F Ratio**								1	-0.03	0.00	-0.01
**H/holds 2+ Cars (%)**									1	-0.07	-0.08
**Giving 2014**										1	0.87
**Giving 2015**											1

Following data pre-processing, Stepwise Regression was applied to the entire data set to identify the most significant variables among 9 demographic variables and one variable for donation amounts in 2014 for 9,410 U.S. zip codes as the input variables to predict donation amounts for 2015.

Stepwise regression is a semi-automated process of model building by successively adding or removing variables based on the t-statistics of their estimated coefficients. This technique starts with a null model and adds the variable with the lowest p-value and continues with forward selection and backward elimination until it reaches to the point where no variable can be added or removed. The alpha-to-enter and alpha-to-remove are often selected as a value between 5% to 15%. We used 5% and 10% as the significant levels for the forward selection and backward elimination in our analysis, respectively.

All data analysis was done in MATLAB environment using a computer with an Intel® Core i5 CPU 2.20 GHz processor and 8 GB RAM. The analysis is described in detail in the following section. The result of the stepwise regression is shown in Table 4.

**Table 4:** Stepwise regression result.

Variable	Coefficient	Standard Error	Status	P-Value
**Intercept**	0.025	0.003	_	<0.0001
**Unemployment Rate (%)**	0.031	0.033	'Out'	0.339
**Average Household Income**	**-0.056**	**0.017**	**'In'**	**<0.0001**
**Poverty Level (%)**	0.019	0.022	'Out'	0.375
**Average Population Age**	-0.001	0.014	'Out'	0.972
**Population**	**0.062**	**0.009**	**'In'**	**<0.0001**
**Whites (%)**	-0.005	0.007	'Out'	0.462
**College Graduates (%)**	**0.063**	**0.010**	**'In'**	**<0.0001**
**Male/Female Ratio**	-0.025	0.042	'Out'	0.551
**Households with 2+ Cars (%)**	-0.013	0.010	'Out'	0.223
**Giving 2014**	**3.996**	**0.025**	**'In'**	**0**

The stepwise regression selected these four variables to build the predictive models: average household income, population, percentage of college graduates, and amount of charitable giving in the previous year. However, the correlation matrix shows that the average household income has high collinearity with the percentage of college graduates. It is crucial to avoid multicollinearity as it can cause unstable coefficient estimation. Variance Inflation Factor (VIF) is a criterion that measures the multicollinearity between variables. The calculation of VIF for *β*_*i*_ estimate is shown in equation.

$${VIF}_{i}=\frac{1}{1-R_{i}^{2}}$$ (2)

In equation, $R_{i}^{2}$ is the coefficient of determination of the regression equation with *X*_*i*_ {\displaystyle X_{i}}on the left hand side, and all other independent variables on the right hand side. The minimum value of VIF is 1, which indicates a model with completely independent variables. A VIF greater than 5 is considered high and can be an evidence of multicollinearity. Table 5. VIF measurement for independent variables Table 5 shows the values of VIF for all independent variables in our model. As we suspected, average household income has a relatively high VIF, meaning that it can be removed from the model without affecting the prediction.

**Table 5:** VIF measurement for independent variables.

Variable	VIF
**Unemployment Rate (%)**	1.93
**Average Household Income**	4.56
**Poverty Level (%)**	3.19
**Average Population Age**	1.61
**Population**	1.39
**Whites (%)**	2.11
**College Graduates (%)**	2.93
**Male/Female Ratio**	1.08
**Households with 2+ Cars (%)**	2.96
**Giving 2014**	1.08

In the results from stepwise regression, giving in the previous year (2014) had the most significant effect, followed by percentage of college graduates. To summarize, the most significant variables among all 10 tested variables were population, percentage of college graduates, and amount of charitable giving in the previous year. Table 5 shows no collinearity in the model with these three variables.

### Predictive modelling approaches

We used the most significant variables identified in the previous section to develop three predictive models based on Multiple Linear Regression (MLR), Artificial Neural Networs (ANN), and Support Vector Regression (SVR). All these techniques use input variables to predict future values of a dependent variable. Their main difference is the way they calculate the weights on the connections between input nodes and output nodes. MLR technique was described in the previous section. Here we describe ANN and SVR approaches in more details.

In MLR, the relationship between two or more explanatory (i.e., independent) variables and a response (i.e., dependent) variable is modeled by fitting a linear equation to the learning data. Table 6 shows a list of notations for MLR technique.

**Table 6:** MLR notations.

Notation	Definition
***i***	Independent variable index
***P***	Number of predictor (Independent) variables
***Y***	Dependent variable (Output)
***β*_0_**	Intercept of the regression model
***β*_*i*_**	Coefficient of Variable 𝑖 in the Regression Model
***x*_*i*_**	Input Variable 𝑖 in the Regression Model (Input)
***ε***	Random error term

The general form of the MLR model is shown in equation.

$$Y=\beta_{0}+\beta_{1}x_{1}+{\ldots +\beta }_{i}x_{i}+\cdots +\beta_{P}x_{P}+\varepsilon$$ (3)

In equation, *β*_0_ is the intercept and *β*_1_,*β*_2_,…,*β*_*P*_ are the corresponding coefficients for independent variables, which will be obtained using the generalized least square method. The error term of the model is denoted by *ε* and the output or dependent variable is shown as *Y*.

The ANN model uses an algorithm to train the network and assign weights to connections between nodes in input layer, hidden layer(s), and output layer. This algorithm can be Back Probagation, Feed Forward, or Feed Backward. In this work, we use a Feed Forward Neural Network (FFNN) which has been widely used in forecasting applications [28]. Table 7 summarizes all the notatios used to explain ANN. All neural network models start with an initial random weight for each connection and improve the weights in their learning process to better predict the output. This process continues until it gets close enough to the output, which is determined by a threshold value.

**Table 7:** ANN notations.

Notation	Definition
***i***	Index unit in input layer or the feature number
***h***	Index unit in hidden layer
***x*_*i*_**	Independent variable i (input)
***P***	Number of predictor (Independent) variables
***net*_*h*_**	Scalar net activation of neuron h in hidden layer
***net*_*o*_**	Scalar net activation of output layer
***f***	Activation function
***o*_*h*_**	Weighted output of neuron h in the hidden layer
***w*_*ih*_**	Weight on the connection from input node i to hidden node h
***w*_*h*_**	Weight on the connection from hidden node h to output node

**Fig 1:**
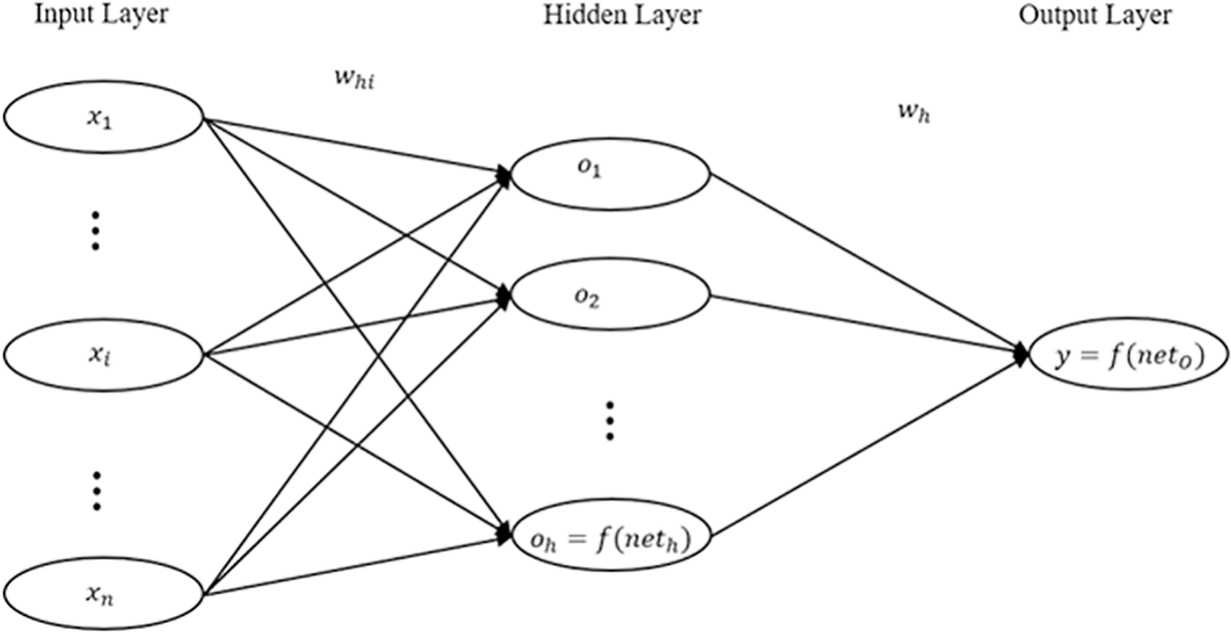
One-hidden layer artificial neural networks model.

Fig 1 shows the structure of a one-hidden layer neural network. Each connection is assigned a weight determined by the activation function of the neural networks. The weight of the connection from input node to the hidden node is denoted as *w*_*ih*_ and the weight of the connection between hidden node and output node is denoted as *w*_*h*_.

With only a few minor differences the SVR uses the same principles as the Support Vector Machine (SVM) for classification which was first developed by Vapnik and Lerner [29]. SVR uses the same concepts to train the model for prediction purposes. The notation used for describing SVR is shown in Table 8.

**Table 8:** SVR notations.

Notation	Definition
***j***	Index unit of *j*^*th*^ data point
***m***	Number of data points
***x*_*j*_**	Input vector for *j*^*th*^ data point
***t*_*j*_**	Target vector for *j*^*th*^ data point
***C***	Cost of not falling inside the SVR tube (Penalty)
***ϵ***	The width of the tube (Acceptable deviation)
$\zeta_{j}^{+}$	Positive deviation from acceptable region for *j*^*th*^ data point
$\zeta_{j}^{-}$	Negative deviation from acceptable region for *j*^*th*^ data point
***w*_*SVR*_**	Regression line weight vector in SVR
***b***	Bias term
***L*_*ϵ*_**	Loss function of the tube with width of *ϵ*
***Gamma***	The Kernel function parameter

**Fig 2:**
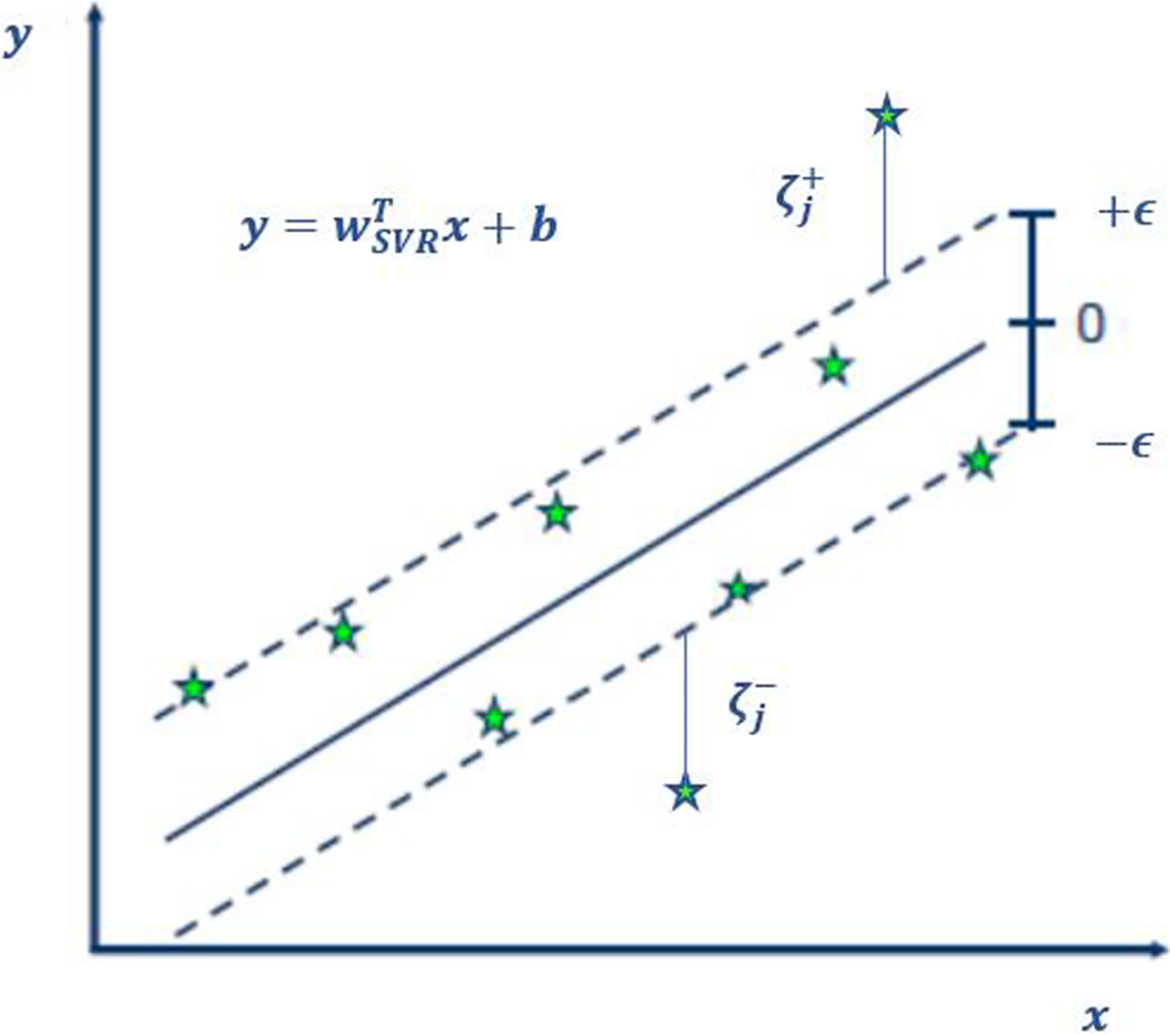
Deviation in SVR [30].

In SVR, a training data set is introduced as {(*x*_1_,*t*_1_),…,(*x*_*j*_,*t*_*j*_),…,(*x*_*m*_,*t*_*m*_)}, where *x*_*j*_ϵ*R*^*n*^ and *t*_*j*_ϵ*R* represent the input vector and the target value, respectively. The main purpose is to obtain a function f(x) that has less deviation than the maximum acceptable deviation *ϵ* from the actual target *t*_*j*_ for all data points in the training data set. SVR aims to determine this function such that the bias and variance trade-offs can be met. This function should be neither so as complex to cause an over-fitting problem nor so simple that it lacks the capability to capture the patterns. To avoid aforementioned problems, a proper value of C is crucial. The maximum deviation from the target value is denoted by *ϵ* shown in Fig 2. The points outside the maximum deviation region are assigned a positive or negative deviation depending on their location.

The regression function is defined in equation. In this expression, *b* is the intercept and $w_{SVR}^{T}$ are the weights attained from SVR. To have a simpler function, smaller sizes of $w_{SVR}^{T}$ are recommended.

$$f(x_{m})=w_{SVR}^{T}x_{m}+b$$ (4)

The loss function defined in equation assigns a penalty if the point is outside the range of the predefined deviation size of *ϵ* shown in Fig 2, which assigns a cost “C” for the points outside of the predefined range.

$$L_{\epsilon }(x_{m},f(x_{m}))=\left\{ \begin{array}{l} 0\;if\;|w_{SVR}^{T}x_{m}+b-t_{m}|\leq \epsilon \\ |w_{SVR}^{T}x_{m}+b-t_{m}|-\epsilon \;otherwise \end{array}\right.\;\forall m$$ (5)

Equation shows the objective function, which assigns a penalty cost, C, if the point is outside of the acceptable range. Equation shows the set of constraints in the SVR model.

$$minimize\frac{1}{2}w_{SVR}^{T}w_{SVR}+C\;\sum_{m=1}^{N}\left(\zeta_{m}^{+}+\zeta_{m}^{-}\right)$$ (6)

Subject to:

$$w_{SVR}^{T}x_{m}+b-t_{m}<\epsilon +\zeta_{m}^{+}\;\forall m$$ (7)

Where $\zeta_{m}^{+}$ and $\zeta_{m}^{-}$ are positive and negative deviations from acceptable region for the *m*^*th*^ pattern, respectively.

## Results

Using MATLAB, the 9410 data rows (zip codes) were randomly divided into 2 separate and exclusive data sets: test (20%) and training (80%). The models were developed using the training data set and tested using the test data set, which was not included in the training part. A 10-fold cross validation technique was used for model validation.

### Multiple linear regression

MLR was applied to the training data set with the selected 3 input variables identified as the most significant variables. Table 9 shows a summary of the results from the MLR technique.

**Table 9:** MLR predictive model parameters.

Variable	Coefficient	Std. Error	t-stat	P-Value	95% Confidence Interval	
**Intercept**	0.021	0.003	7.344	<0.00001	0.016	0.027
**Population**	0.071	0.001	7.254	<0.00001	0.051	0.090
**College Graduates (%)**	0.038	0.008	4.757	<0.00001	0.022	0.053
**Giving 2014**	3.903	0.028	141.228	0	3.849	3.957

The Analysis of Variance (ANOVA) table for this model shown in Table 10 confirms that this model is significant at 5% significance level.

**Table 10:** ANOVA table for MLR predictive model.

Source	DF	Sum of Squares	Mean Square	F-Value	P-Value
**Regression**	3	290.291	96.764	7310.093	0
**Error**	7524	99.595	0.013		
**Total**	7527	389.886			

**Fig 3:**
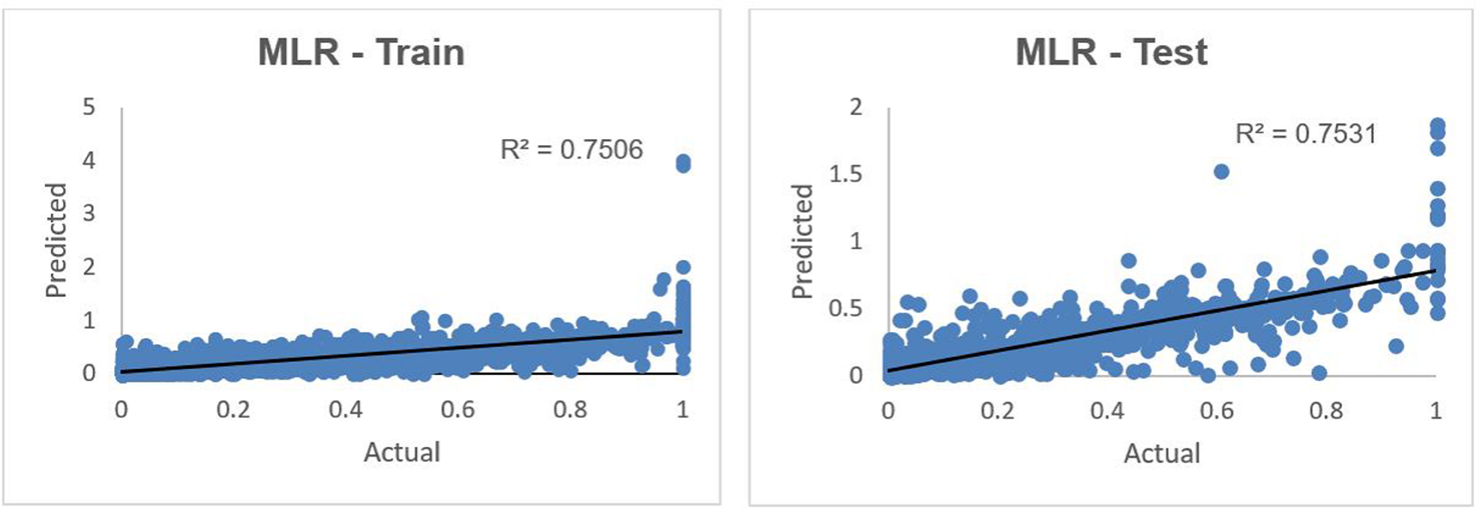
Predicted vs. actual 2015 donations using MLR on training and test data sets.

This model was tested using the test data set. Fig 3 shows the predicted versus actual donations in 2015 in a normalized scale using MLR technique.

### Artificial neural networks

The same training data set was used to train and build a model using ANN. This model has 1 hidden layer. The number of neurons in the hidden layer varies from 2 to 10, demonstrating that 2 neurons minimized the percentage of residual variance. In this study, we considered 1 hidden layer. The network with 1 hidden layer and 2 neurons in that single hidden layer gives the best ANN model among all the tested networks with learning rate of 0.001.

Table 11 shows the characteristics of our ANN model. Logistic and Linear activation functions have been adopted for the input and hidden layers, based on a trial and error, to produce less error.

**Table 11:** ANN predictive model parameters.

Layer	Neurons	Activation Function	Min. Weight	Max. Weight
**Input**	3			
**Hidden**	2	Logistic	-0.379	3.640
**Output**	1	Linear	-1.320	2.970

**Fig 4:**
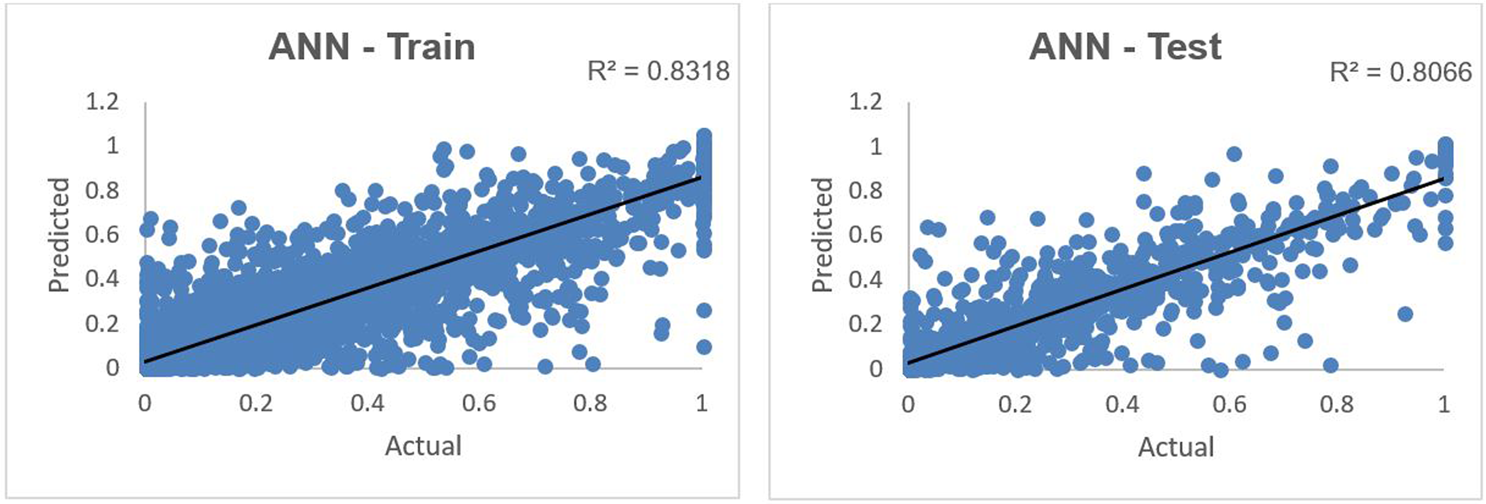
Predicted vs. actual 2015 Giving using ANN on training and test data sets.

The predicted versus actual donations in 2015 in a normalized scale using the ANN technique is shown in Fig 4.

### Support vector regression

SVR is the the other machine learning technique that we use to build a predictive model for 2015 donations. The same training and test data sets are used in this approach. Table 12 demonstrates the parameters of the best model we could capture on this data set.

**Table 12:** SVR predictive model parameters.

Parameter	Value
**Epsilon (*ϵ*)**	0.018
**C**	1.0
**Gamma**	4.51
**Bias**	-0.113

**Fig 5:**
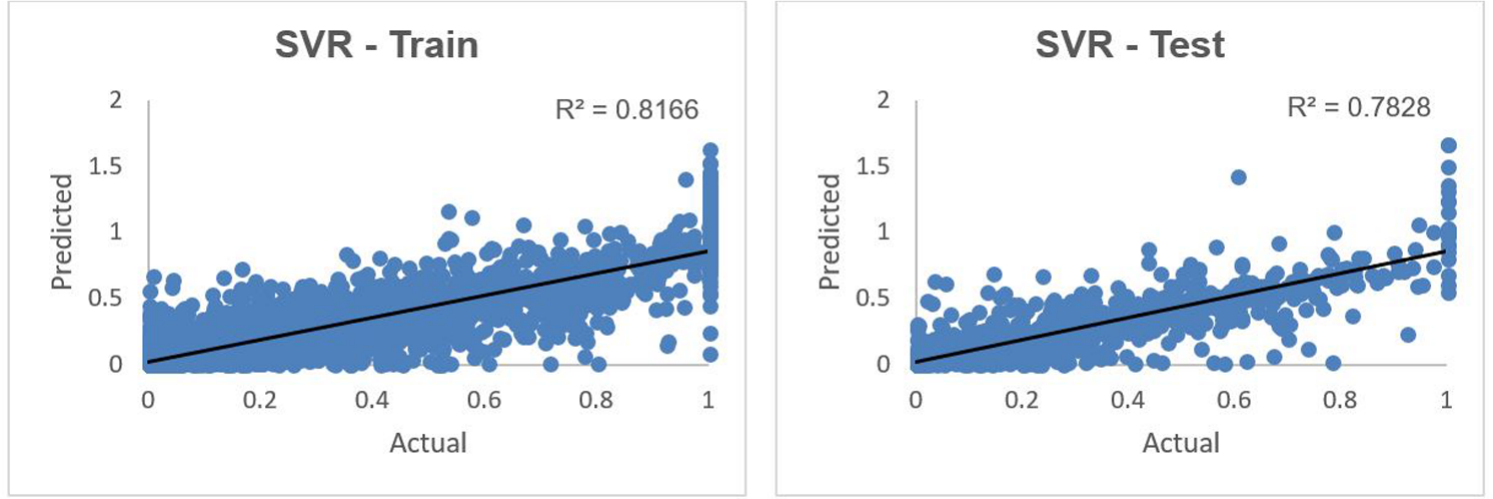
Predicted vs. actual 2015 giving using SVR on training and test data sets.

Fig 5 shows the results from the SVR predictive model on the training and test data sets, comparing the predicted vs the actual 2015 giving in normalized scale.

## Discussion

The difference between the actual (target) and the predicted value is the forecasting error which represents the accuracy measure. To evaluate the performance of the applied models in this study, some accuracy measures have been calculated as follows:

1. Symmetric Mean Absolute Percentage Error (SMAPE) is calculated using equation. SMAPE is an alternative criterion for Mean Absolute Percentage Error when there are zero values for giving.
  $$SMAPE=\frac{2}{n}\sum_{i=1}^{n}\left|\frac{x_{i}^{P}-x_{i}^{A}}{x_{i}^{P}+x_{i}^{A}}\right|$$ (8)
2. Mean Absolute Error (MAE) is calculated using equation. A MAE that approaches zero is an indication of the model’s high accuracy.
  $$MAE=\frac{\sum_{i=1}^{n}\left|x_{i}^{P}-x_{i}^{A}\right|}{n}$$ (9)
3. Root Mean Square Error (RMSE) is calculated using equation. Small RMSE values also denote good performance on the part of the model.
  $$RMSE=\sqrt{\frac{\sum_{i=1}^{n}{\left(x_{i}^{P}-x_{i}^{A}\right)}^{2}}{n}}$$ (10)
4. Normalized Root Mean Square Error (NRMSE) is calculated using equation. A NRMSE value close to 1 indicates a poor model performance, whereas value close to 0 shows a good model performance.
  $$NRMSE=\sqrt{\frac{\sum_{i=1}^{n}{\left(x_{i}^{P}-x_{i}^{A}\right)}^{2}}{\sum_{i=1}^{n}{\left(x_{i}^{A}\right)}^{2}}}$$ (11)
5. Mean Square Error (MSE) is calculated using equation. The value of MSE depends on the scale of data but we report the MSE values on the normalized data for easier comparison.
  $$MSE=\frac{1}{n}\sum_{i=1}^{n}{\left(x_{i}^{P}-x_{i}^{A}\right)}^{2}$$ (12)

Where $x_{i}^{P}$ is the predicted value and $x_{i}^{A}$ is the actual value of giving for the *i*^*th*^ observation.

Using and comparing several accuracy measures alongside each other enables us to better evaluate the results, as each accuracy measure has its own advantages and limitations and there is no single measure that is universally applicable under all conditions [31]. The values of all accuracy measures for all three models is shown in Table 13.

**Table 13:** Test results comparison of all three techniques.

Criteria	Definition	MLR	ANN	SVR
**SMAPE**	Symmetric Mean Absolute Percentage Error	0.829	0.765	0.759*
**MAE**	Mean Absolute Error	0.067	0.055*	0.057
**RMSE**	Root Mean Squared Error	0.111	0.098*	0.105
**NRMSE**	Normalized Root Mean Square Error	0.396	0.350*	0.374
**MSE**	Mean Squared Error	0.012	0.010*	0.011
**Residual**	Unexplained variance after model fit	23.294	18.268*	20.837
**R^2^**	Proportion of variance explained by model	0.753	0.807*	0.783
**Max Err**	Maximum error	0.933	0.611*	0.877
**R**	Correlation between actual and predicted	0.868	0.898*	0.885

*- Best performing technique

As Table 13 shows, ANN outperforms SVR and MLR in predicting the charitable giving using the three significant variables comparing R-Squared (R^2^) and error terms on this data set. According to this Table 9 measuring criteria show that ANN performs slightly better in predicting the U.S. charitable giving.

### Conclusions and future work

In this paper, we studied the effect of 10 factors (population, personal income, education level, unemployment rate, poverty, and charitable giving in a previous year) on the receipt of charitable giving. Stepwise regression identified the four most influential variables among these 10 tested variables to be average household income, population, percentage of college graduates, and giving amounts for the previous year. To avoid dealing with multicollinearity, average household income was excluded as it was highly correlated with percentage of college graduates. All variables have positive relationship with donation. Previous year donation is the most highly correlated factor and percentage of college graduates is the least correlated. Three forecasting models were developed using MLR, ANN, and SVR and tested on a data set. To compare, 9 criteria measures were calculated for the results of each model. All three models are capable of predicting the amount of future donation in a given region with good accuracy, however, ANN outperforms SVR and MLR in most cases.

Moving forward, we plan to expand our study using other machine learning techniques, and by developing comparative studies to find potential ways to improve our suggested models. There are many reasons why being able to forecast charitable giving will help organizations better plan. For example, the occurrence of natural and man-made disasters may affect giving both in positive and in negative ways [32]. As an instance, national level disasters could potentially decrease giving, while a regional natural disaster may increase giving from those unaffected. After the terrorist attacks of September 11, 2001 (New York City, Washington D.C., Somerset County, PA), 65% of US household made charitable contributions [33]. The largest not-for-profit recipient organization received approximately $2 billion USD by the end of 2001. This was the largest giving in recorded US history. Although a blessing, such a large unexpected and rapid charitable influx can create its own complications for the receiving organization as a lack of preparedness, may potentially result in mismanagement of the contributions [34]. This example illustrates why charitable organizations, especially those involved in disaster relief efforts, need to be able to accurately forecast charitable giving to promote more equitable and efficient use of resources.

We plan to study the effects of natural or man-made disasters on the donor’s behavior, and to develop models and evaluate them using the recent disasters, such as hurricane Sandy. Furthermore, future research could identify and incorporate national economic factors in the predictive models.
